# Protein Glycation in Diabetes as Determined by Mass Spectrometry

**DOI:** 10.1155/2013/412103

**Published:** 2013-03-13

**Authors:** Annunziata Lapolla, Laura Molin, Pietro Traldi

**Affiliations:** ^1^Department of Medicine, Padova University, Via Giustiniani 2, I35100 Padova, Italy; ^2^National Council of Researches, Institute of Molecular Sciences and Technologies, Corso Stati Uniti 4, I35127 Padova, Italy

## Abstract

Diabetes is a common endocrine disorder characterized by hyperglycemia leading to nonenzymatic glycation of proteins, responsible for chronic complications. The development of mass spectrometric techniques able to give highly specific and reliable results in proteome field is of wide interest for physicians, giving them new tools to monitor the disease progression and the possible complications related to diabetes, as well as the effectiveness of therapeutic treatments. This paper reports and discusses some of the data pertaining protein glycation in diabetic subjects obtained by matrix-assisted laser desorption ionization (MALDI) mass spectrometry (MS). The preliminary studies carried out by *in vitro* protein glycation experiments show clear differences in molecular weight of glycated and unglycated proteins. Then, the attention was focused on plasma proteins human serum albumin (HSA) and immunoglobulin G (IgG). Enzymatic degradation products of *in vitro* glycated HSA were studied in order to simulate the *in vivo* enzymatic digestion of glycated species by the immunological system leading to the highly reactive advanced glycation end-products (AGEs) peptides. Further studies led to the evaluation of glycated Apo A-I and glycated haemoglobin levels. A different MALDI approach was employed for the identification of markers of disease in urine samples of healthy, diabetic, nephropathic, and diabetic-nephropathic subjects.

## 1. Introduction 

Diabetes is usually considered as a disease related to glucose dysmetabolism. In particular, type 1 diabetes is a chronic disease related to metabolism of carbohydrates, fats, and proteins, caused by the lack of insulin. It results from the marked and progressive inability of the pancreas to secrete insulin, due to autoimmune destruction of the beta cells. On the other hand, type 2 diabetes is caused by islet beta cells being unable to secrete adequate insulin in response to varying degrees of overnutrition, inactivity, obesity, and insulin resistance. Nowadays, the burden of diabetes is enormous, due to its increasing global prevalence and the occurrence of chronic complications affecting many tissues (retinopathy, nephropathy, neuropathy, and cardiovascular disease) reflecting in high direct and indirect costs [1].

This view may be seen somehow reductive, considering that the side effects of the previous mechanisms are at systemic level, and, taking into account the high complexity of the biological environment, it necessarily reflects on a high number of different pathological pathways, catalyzed by the glucose dysmetabolism. In this context, considering the Maillard reaction pattern [2], proteins seem to be at first sight the target of the glucose molecules circulating at high level in diabetes, and only some papers gave contradictory results about the reactivity of sugar with respect to DNA [3, 4]. 

The nonenzymatic reaction between proteins and sugars (mainly glucose and fructose) leads to glycated proteins which, depending on the number of glucose molecule condensed on them, would exhibit a different functionality. This aspect can be considered a *rationale* for the activation of new pathologies. As an example, consider the role of human serum albumin (HSA) as transport protein, in the case of its extensive glycation, the active sites responsible for this function would not be still available and the activity of this protein would be deeply impaired. The same can be considered for immunoglobulins, which play a fundamental role at immunosystemic level. These two examples have been given because we investigated on these aspects some years ago, and the related data will be discussed later [5, 6]. These general considerations are a good starting point to recognize the importance of proteomic studies in diabetes field. In this paper, the results obtained by matrix-assisted laser desorption ionization mass spectrometry (MALDI-MS) in the study of protein glycation are reported and discussed, with the aim to give descriptions of limits and power of the technique.

## 2. Proteomics Studies by Mass Spectrometry

The classical approach in proteomics usually consists in the separation of the different proteins contained in the biological substrate, the digestion of the separated proteins, and the analysis of the digestion products by mass spectrometry. Of course as specific is the mass spectrometric approach, as valid are the results obtained for the structure identification of the proteins of interest [7]. For this aim, two different routes can be followed. The first is a chemical approach, based on the selection of the protonated molecular species ([*M*+*H*]^+^) of the tryptic digestion product and on the study of its fragments obtained by collisional phenomena (MS/MS) [8]. The second approach is based on physical measurement of the accurate masses of either precursor ion or its fragmentation products [9]. To obtain the protein identification, these data are used for an interaction with protein databases. MS-Fit, MOWSE, Prot-ID, Expasy tools, and Peptide search are some of the database-search programs that can be used to identify the protein subjected to enzymatic digestion [8, 10]. The molecular masses of peptides in the query are matched against the theoretical peptide-mass values created by *in silico* digestion of each protein entry in the database with the specific protease that was used in the experimental step. In a typical search algorithm, after selection of the cleavage database, criteria of the data search (species, mass-matching tolerance, approximate upper value of the molecular mass of the protein, pI value of the protein, minimum number of matches, number of cleavage sites missed by enzymatic digestion, and type of cystein modification) are defined. Considering the list of experimentally obtained [*M*+*H*]^+^ values of the peptides, the output of the search gives a list of most likely candidates. The sequence with the highest score has the highest probability to individuate the protein of interest. The selectivity of the search can be increased by keeping the mass-matching tolerance low (<3 ppm) and increasing the mass measurement accuracy (±0.5 Da), requirement easier met by acquiring data with delay-extraction time of flight mass spectrometry (TOF-MS) and Fourier transform ion cyclotron resonance mass spectrometry (FT-ICR-MS). MASCOT and SEQUEST instead are the most widely used search algorithms to identify proteins on the basis of the MS/MS sequence ion data [10]. In these search engines, the pattern of fragment ions observed is matched with the fragment ion pattern calculated theoretically from the database entries. SEQUEST algorithm simplifies the acquired MS/MS spectrum, identifies amino acid sequence in the database with the measured mass of the selected peptide ion, and predicts the fragmentation pattern that is expected for each sequence. The spectra are subjected to Fourier transform, and each virtual spectrum is matched with the experimentally observed MS/MS spectrum to produce a cross-correlation score. 

In our studies, we employed generally a different approach, based on the determination of molecular weight of glycated proteins and its comparison with the unglycated ones. At the beginning, we focalized our attention on circulating proteins for their easy availability, and we employed MALDI-MS, due to its ability to give a direct information on molecular species, even if present in complex mixture. 

## 3. Studies on *In Vitro* Protein Glycation

**Figure 1:**
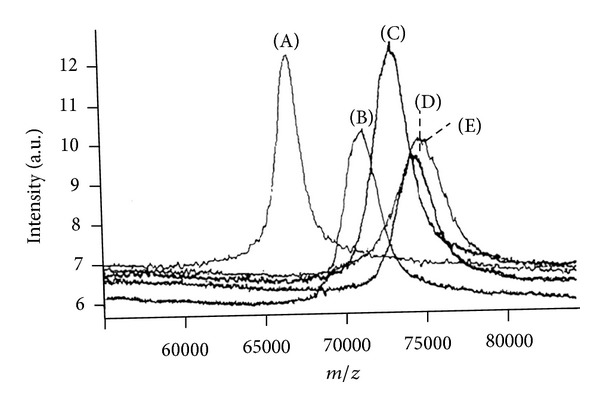
MALDI spectra of BSA incubated with 2 M glucose at concentration (pH 7.5; 37°C) recorded at different incubation times: (A) incubation time, 0 days, molecular mass 66429 Da; (B) incubation time, 7 days, molecular mass 71103 Da; (C) incubation time, 14 days, molecular mass 73099 Da; (D) incubation time, 21 days, molecular mass 74279 Da; (E) incubation time, 28 days, molecular mass 74682 Da. From [11], by kind permission of John Wiley and Sons through RightsLink, License no. 3064281057207.

The first work based on this approach was carried out at the beginning of the 90s and, at that time, the validity of MALDI-MS in protein glycation studies had to be proved. For this reason, a series of preliminary investigations were carried out on *in vitro* glycated different proteins [11–14]. Typical results obtained by this approach are reported in Figure 1. By incubating bovine serum albumin (BSA) in pseudophysiological conditions (phosphate buffer 0.05 M, pH 7.5, 37°C) with glucose (concentration 2 M), a clear increase of the mean molecular weight is observed by increasing the incubation time (0, 7, 14, 21, 28 days), proving the occurrence of glucose condensation on the protein. It must be considered that according to the Maillard reaction pathway, this mass increase is the result of an equilibrium between glucose condensation on *ε*-amino groups of lysine belonging to the protein chain and the release of active compounds in the intermediate step of the reaction itself. 

## 4. Studies on *In Vivo* Glycated Proteins

**Figure 2:**
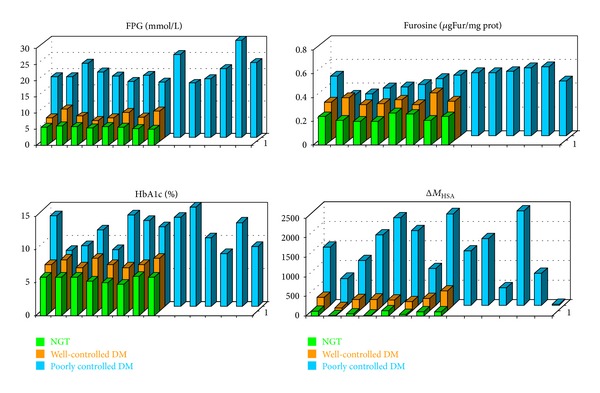
Fasting plasma glucose (FPG), furosine, HbA1c levels, and HSA Δ*M* values obtained for healthy subjects (first line), well-controlled diabetic patients (second line), and poorly controlled diabetic patients (third line).

**Figure 3:**
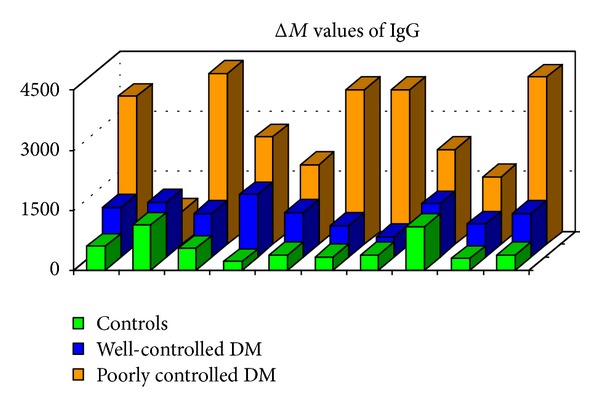
Δ*M* values of IgG obtained for healthy (first line), well-controlled diabetic patients (second line), and poorly controlled diabetic patients (third line).

Once confident on the validity of the results achievable by MALDI-MS, our attention was focused on HSA and immunoglobulin G (IgG) from diabetic patients. For this aim, three different population of subjects (homogeneous in age and sex): eight healthy subjects (mean age ± standard deviation (SD) of 57 ± 9 years), eight well-controlled, non-insulin-dependent, diabetic patients (mean age 60 ± 12 years, mean disease duration 13 ± 9 years), and fourteen badly controlled, non-insulin-dependent diabetic patients (mean age 63 ± 7 years, mean disease duration 12 ± 8 years), were considered. In the last two cases, a mass increase of the molecular species related to HSA and IgG was observed, and such increase (Δ*M*) was easily related to the minimum number of glucose molecules (*n*) condensed on the protein [15, 16]. Considering that the condensation of a glucose molecule leads to a mass increase of 162 Da, *n* can be calculated by *n* = Δ*M*/162. It is to emphasized that *n* represents the minimum number of glucose molecules: in fact, as shown by the Maillard reaction pattern, the condensed glucose molecules can undergo a series of dehydration/oxidation reactions leading to species at lower molecular weight. In Figure 2, the data obtained on HSA for the previous populations are compared with that obtained by “classic” metabolic control parameters as fasting plasma glucose, HbA1c, and furosine (Table 1). In the case of badly controlled patients, a number of glucose molecules condensed on the protein ranging from 4 to 15 are observed. The same approach was applied to investigate the IgG glycation, and, in the case of badly controlled diabetic patients, a number of glucose molecules ranging from 7 to 28 were present (Figure 3).

What is at first sight evident is that while the values of the common metabolic control parameters are quite uniform inside each class of subjects, in the case of Δ*M* values obtained by mass spectrometric measurements, a wide intraclass variation is present. These results can be explained by hypothesizing the occurrence of two different mechanisms: the first indicates a different subject-depending protease activity which in some cases leads to a rapid glycated protein digestion; the second could suggest the presence, for some patients, of an enzymatic system able to reverse the nonenzymatic glycation reaction. In this frame, fructosamine 3-kinase gene, located on chromosome 17q25.3 and organized in six exons, codes a 34 kDa protein expressed in every human tissue and whose greatest expression is in diabetes susceptible organs, such as kidney, heart, and nervous tissue [17]. This enzyme is involved in the reverse of nonenzymatic glycation phosphorylating fructoselysine residues to fructoselysine-3-phosphate (FL3P) at the expense of ATP [18]; this destabilizes the fructos-amine linkage, leading to spontaneous decomposition of FL3P to lysine, 3-deoxyglucosone, and inorganic phosphate [19]. Very few studies have been reported regarding genetic variants in FN3K and its enzymatic activity; Delpierre and co-workers reported an association between the erythrocyte FN3K enzymatic activity and some polymorphisms in the FN3K gene on a Belgian subpopulation [20]. Then, the polymorphism c.900 C/G (rs1056534) located in exon 6 and lower HbA1c levels in type 2 diabetes patients have been shown together with a tardive onset of the disease [21]. A more recent study has identified two new mutations linked to T2DM and to female gender; furthermore, additional variants within FN3K gene are here reported adding new useful information to the possible role of FN3K in diabetes [22]. Unfortunately, fructosamine 3-kinase was not genotyped in the subjects under study, and consequently the previous rationale must be considered only reasonable.

## 5. Identification of Advance Glycation End-Product (AGE) Peptides

**Figure 4:**
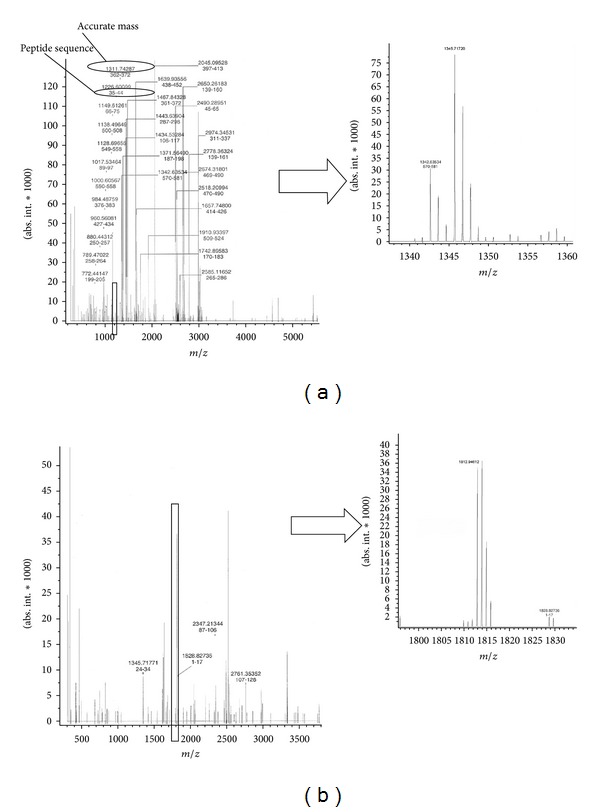
(a) FTMS ESI spectrum of the enzymatic digestion products of untreated HSA; (b) FTMS ESI spectrum of the enzymatic digestion products of *in vitro* glycated HSA.

**Figure 5:**
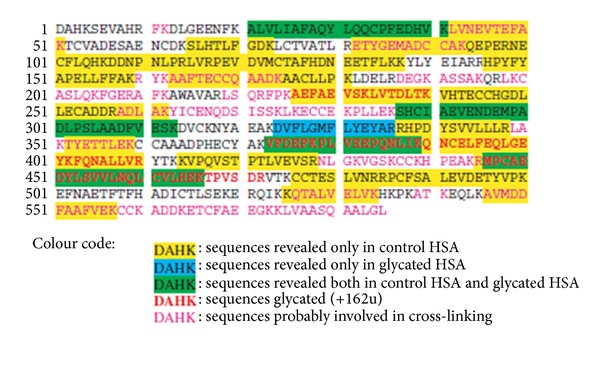
Peptides identified by accurate mass measurements based on HSA sequence. From [23], by kind permission of John Wiley and Sons through RightsLink, License no. 3064270300383.

A glycated protein is considered by the immunological system an “undesired” species, and consequently its enzymatic digestion is activated. It must be considered that this process is unfavored; in fact, the glycated proteins are more difficult to be digested, due to steric effects induced by the condensed glucose, which do not allow the enzyme action on the protein chain. Furthermore, the glycated peptides released by this digestion (called AGE peptides) exhibit a high reactivity with respect to other circulating or tissue proteins, leading to structure modification more severe than those due to simple glucose. To study this aspect, a series of investigations has been carried out by accurate mass measurement obtained by Fourier transform mass spectrometry (FTMS) on the enzymatic digestion products of HAS [23]. Clear differences were observed between the digestion mixture of glycated and unglycated serum albumin, and in the former case, possible glycated peptides belonging to the AGE peptide class were identified. As an example of the power of this method, the spectra of these two mixtures are reported in Figure 4, and by the highly accurate mass value determination, peptides originating by digestion of HSA and glycated HSA have been identified (see Figure 5).

**Figure 6:**
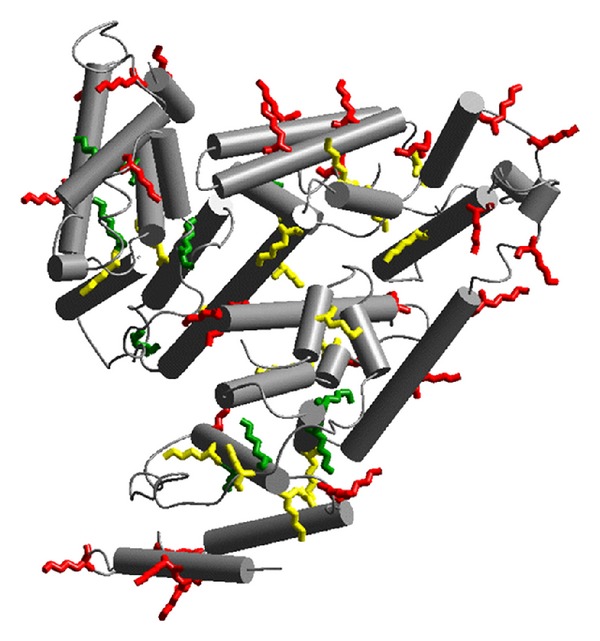
Most solvent-exposed lysine residues, color-coded according to their range of fractional solvent accessible surfaces (red: 0.5–1, most exposed; yellow: 0.3–0.5, less exposed; green: 0.1–0.3, buried). From [24], by kind permission of Springer through RightsLink, License no. 3064270675691.

**Figure 7:**
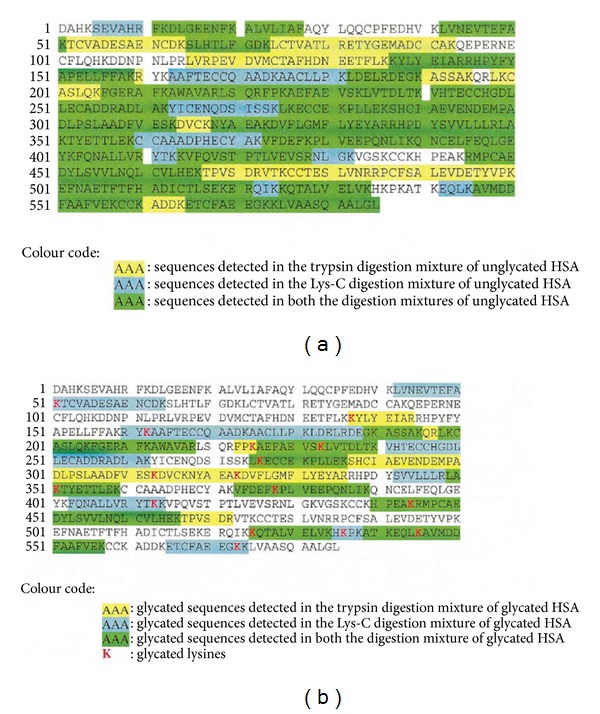
(a) Peptides identified by ESI/LC/MS analyses of unglycated HSA; (b) glycated peptides identified by ESI/LC/MS analyses of glycated HSA. From [24], by kind permission of Springer through RightsLink, License no. 3064270675691.

In a further study, MS/MS experiments were carried out on the peptide mixtures obtained by HSA and glycated-HSA by the action of two different enzymes [24]. This investigation allowed to establish that the most privileged glycation sites in HSA are ^235^K, ^276^K, ^378^K, ^545^K, and ^525^K. These experimental data were in good agreement with the fractional solvent accessible surface values calculated by molecular modeling (Figure 6). Also, in this case, the peptide mapping was obtained (Figure 7), in agreement with both experimental and theoretical data. 

## 6. Investigation on Haemoglobin Glycation Process

**Figure 8:**
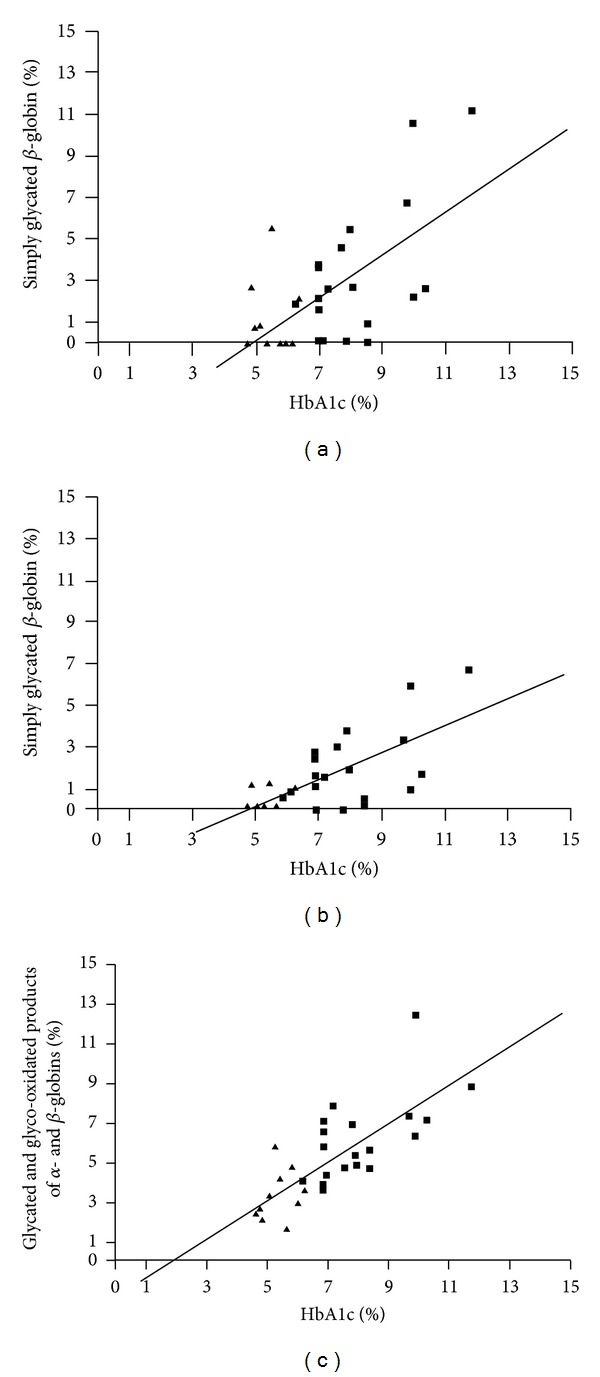
Plots of HbA1c values versus: *β* globin glycation levels (a); *β* + *α* globins glycation levels (b); glycated and glyco-oxidated *β* and *α* globins levels (c).

Considering the high specificity data obtained in the study of *in vivo* glycation of HSA and IgG, a further investigation was addressed to the *in vivo* glycation of haemoglobin. The glycation level of haemoglobin is usually employed for the assessment of the mean glycation level present in the subject. Actually, considering that the half-life of haemoglobin is 120 days, the measurement of glycated haemoglobin can provide valid information on the “glycation stress” experienced by the subject during the protein life. The *α*-amino group of the *β* chain is considered, on the basis of *in vitro* experiments, the most reactive site, and consequently the measurement of glycation level of haemoglobin is considered to be related to the glycation level of *β* globin. For this evaluation, chromatographic procedures able to separate glycated and unglycated globins have been developed and are currently employed to measure what is considered the glycation level of *β* globin (HbA1c). However, it is to emphasize that the chromatographic conditions employed for the clinical test are unable to separate the two globins (*α* and *β*). When globin fractions of subjects with diabetes were analyzed by MALDI [25–28], besides the signals due to protonated *α* and *β* globins (at *m*/*z* 15126 and 15866, resp.), further peaks were identified at higher mass values, showing the occurrence of glycation and glycoxidation of both *α* and *β* globins. The glycation levels were easily calculated by the abundance ratio of the peaks corresponding to glycated and unglycated species. Thus, on the contrary of what is usually thought, both globins are glycated, and consequently the HbA1c value has necessarily a meaning different from that usually considered to be correct. As a matter of fact, when the HbA1c values measured by the HPLC method are plotted with respect to the glycation level of *β* globin obtained by MALDI for twenty healthy subjects (mean age ± SD: 58 ± 5 years, fasting plasma glucose mean value: 90 ± 4 mg/dL; HbA1c mean value: 5.5 ± 0.5%) and thirty non-insulin-dependent diabetic patients (mean age ± SD: 63 ± 6 years; mean disease duration ± SD: 12 ± 5; fasting plasma glucose mean value: 196 ± 67 mg/dL; HbA1c mean value: 8.8 ± 1.7%), a straight line not passing through the origin is obtained (Figure 8(a)), demonstrating that the HbA1c value is not simply due to *β* globin glycation. The best linear relationship (Figure 8(c)) has been obtained by plotting the HbA1c values with the total level, obtained by MALDI, of glycated and glycoxidated *α* and *β* globins. The peaks due to nonglycated and simply glycated globins and those due to glycated/oxidated species are well defined. Among them is a *β*-globin containing a glyoxal moiety (*β* + 57) at *m*/*z* 15921 Da. However, there were some clear differences between different patients. Although glycated *α*- and *β*-globins were present, a higher number of glyco-oxidated molecules were also detectable, including the species *α* globin + 5-hydroxymethylfuran (*m*/*z* 15225 Da). These results are consistent with the data reported by Hempe and coworkers [29], who postulated the existence of both high and low haemoglobin glycation phenotypes. Our results indicate that different subjects have a different tendency to oxidation processes occurring after globin glycation. In order to further explore this concept, twenty patients with type 2 diabetes with and without chronic complications were studied to evaluate the possible relationship of the information obtained by MALDI/MS and the actual clinical condition [28]. Interestingly, the presence or absence of chronic clinical complications affected the slope of the linear regression line. We suggested that the differences observed between patients with and without clinical complications are also due to a different individual tendencies for oxidation (as observed for glycation) and/or different oxidation kinetics related to behavioural and environmental factors. 

## 7. Studies of Glycation of Lipoprotein Apo A-I

**Figure 9:**
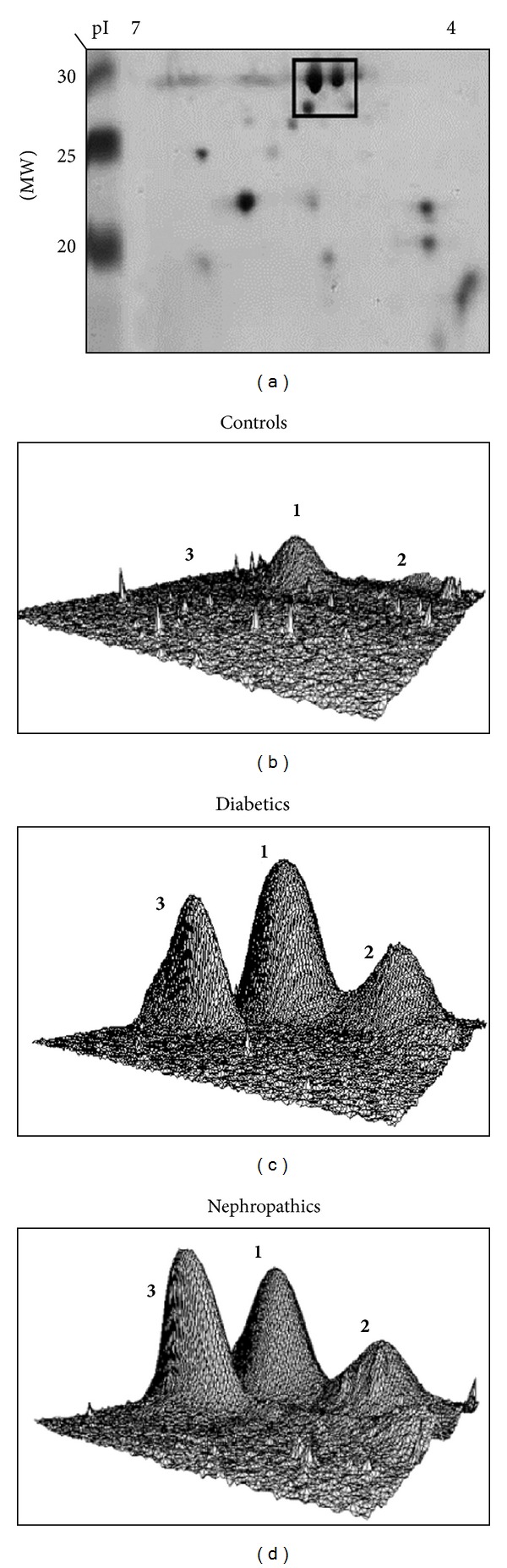
(a) 2D and 3D plots of 2-DE protein spots **1**,** 2**, and **3** (Apo A-1, glycated Apo A-1, and RBP, resp.) of (b) control, (c) diabetic, and (d) nephropathic subjects. From [30], by kind permission of Wiley and Sons through RightsLink, License no. 3064270990822.

Lipoprotein Apo A-I constitutes 70% of the Apolipoprotein content of HDL and acts as acceptor for the transfer of phospholipids from peripheral tissues, and it transports cholesterol in the liver and other tissues for the excretion and steroidogenesis. Its possible glycation would lead in principle to a damage of its functionality, activating atherosclerotic vascular disease. Atherosclerotic vascular disease is a major complication of diabetes, and among the known risk factors for atherosclerosis, (such as hyperlipoproteinemia, obesity, hypertension, hyperinsulinemia, and inflammation), low levels of HDL play an important role. The possible posttranslational modification of Apo A-I due to nonenzymatic glycation processes was investigated by MALDI-MS and 2D-gel electrophoresis [30]. The pool samples from controls, diabetic, and nephropathic subjects were firstly analyzed by 2D-gel electrophoresis, and some interesting results, summarized in Figure 9, were obtained. A significant difference among the three groups is clearly visualized in the 3D views of the area of interest. As can be easily observed, while in the case of healthy subjects practically only one peak is present in the 3D plot, in the case of diabetic and nephropathic patients, three different peaks are clearly detectable in the same region. Enzymatic digestion of the differentially expressed spots followed by MALDI analysis showed with high statistical confidence (*P* value from 3.6 × 10^−24^ to 1 × 10^−7^) that spots 1 and 2 correspond to Apo A-I and spot 3 to retinol-binding protein (RBP), indicating a significant overexpression of these proteins in the examined pathological cases. It is to emphasize that there has been controversy in the literature regarding the role of RBP in the development of insulin resistance and diabetes. In a study on RBP-4 in human obesity [31], it was shown that circulating RBP-4 levels were similar in normal weight, over-weight, and obese women, while in adipose tissue, it was positively correlate with GLUT 4 expression. In a further study [32] on the RBP4 concentrations in response to short-term overfeeding in normal-weight, overweight, and obese men, no differences were found between the two groups, and furthermore baseline RBP4 was negatively correlated with changes in insulin resistance in normal-weight subjects.

In particular, the enzymatic digestion of spot 2 followed by MALDI analysis and data evaluation shows that this protein corresponds to glycated Apo A-I, present in a much smaller extent also in the case of normal subjects. Aldente and Profound PMF tools were used to identify the digested proteins analyzed, while the algorithm GlycoMod was employed to identify the modified glycated peptide. Modified peptide sequences were confirmed by the postsource decay (PSD) approach [33]. In particular, the InSpecT software available online, based on the tag sequencing approach, was employed to obtain the peptide sequence from PSD fragmentation spectra. Each modified sequence was assigned by means of a statistical score, expressed in *P* value, provided by the software. The glycation sites have been individually identified by the analysis of digestion fragments. In the case of spot 2, new mono- and diglycated peptides are evidenced (mass accuracy between 4 and 58 ppm), proving the occurrence of *in vivo* glycation processes, confirmed by MS/MS. These data imply that in plasma samples of both diabetic and nephropathic subjects, glycated Apo A-I is present in an abundance comparable to that of unglycated protein. Furthermore, considering equal amounts of plasma sample, both unglycated and glycated proteins are overexpressed in these groups in comparison with control subjects. The data obtained indicate that the evaluation of Apo A-I, glycated Apo A-I, and RBP can be considered a valid diagnostic tool to assess the metabolic state of patients with diabetes and/or nephropathy. In fact, while glycated Apo A-I levels can be related with the glyco-oxidation stress experienced by the patients during the half-life of the protein, the change in functional capacity of the protein due to glycation necessarily reflects a different cholesterol transport efficiency. This aspect could provide a rationale in considering some of the long-term diabetic complications. It is to emphasize that the same trend was observed for end-stage renal disease patients, originating by a different mechanism related to the efficiency of glycated Apo A-I clearance. These results can explain the occurrence of macrovascular disease in both types of patients. The increase of RBP levels in the case of patients must be related to two different mechanisms, typical of the kind of disease. In subjects with diabetes, obesity may account for the overexpression of RBP, while in the case of patients with nephropathy, it can be related to its impaired excretion due to tubular damage. 

## 8. Biomarker Assessment in Chronic Kidney Disease

Kidney disease is one of the chronic diabetic complications counting for a wide social and medical engagement. It represents a major healthcare problem in all Western countries [34]. Albuminuria is a well-known predictive marker of progression of renal disease in diabetes mellitus [35, 36] and is currently utilized in monitoring renal function in these patients. However, some controversy exists about its sensitivity and specificity [37, 38]. Then, the development of new analytical methods effective in monitoring renal function is surely of wide interest, giving to the physician new biochemical information on the possible pathological mechanisms present and/or in development. Early identification of patients at risk to develop renal complications could be important in order to apply medical intervention able to prevent further progression of the disease [39], thus saving the quality of life and avoiding the costs related to the treatment of end-stage renal disease that can occur in these patients. Recently, Rao et al. carried out an extensive study for the identification, by the proteomic approach, of possible biomarkers of diabetic nephropathy [40]. For this aim, urine samples were collected from 33 subjects with type 2 diabetes and with different microalbuminuria levels and from 9 healthy control subjects. The analytical approach was the classical one: urine proteins were subjected to 2D differential in-gel electrophoresis (DIGE), stained with Coomassie Blue. The individual spots were cut from the gel, distained, and digested with trypsin. The tryptic peptides were analyzed by LC/MS/MS (quadrupole-time of flight (Q-TOF)). The data so obtained were analyzed by Protein-Lynx Global Server and by *de novo* sequencing using a PEAKS algorithm combined with the OpenSea alignment algorithm. This approach led to the identification of 195 protein spots representing 62 unique proteins. They belong to different functional groups (e.g., cell development, cell organization, metabolism, transduction, and defence response). The comparison between control subjects and diabetic patients put in evidence a different expression of several proteins. In particular by spot volume quantification, seven proteins upregulating with increasing albuminuria and four proteins downregulating with it were found.

More recently, a different method, capillary electrophoresis (CE), has been shown to be highly specific and effective for this kind of investigation. Capillary electrophoresis coupled to mass spectrometry (CE-MS) allowed the identification of specific urinary peptide biomarkers of chronic kidney disease (CKD). In a recent multicentre study [41], 609 urine samples from 230 patients with various biopsy-proven CKDs and 379 controls were analyzed using CE-MS to establish a CKD specific biomarker pattern consisting of 273 urinary peptides. This model was subsequently validated in a blinded test set of 280 samples yielding 97.8% sensitivity and 85.5% specificity. Most of the CKD biomarker peptides were found to be fragments of collagen, uromodulin, and some different blood protein [40].

**Figure 10:**
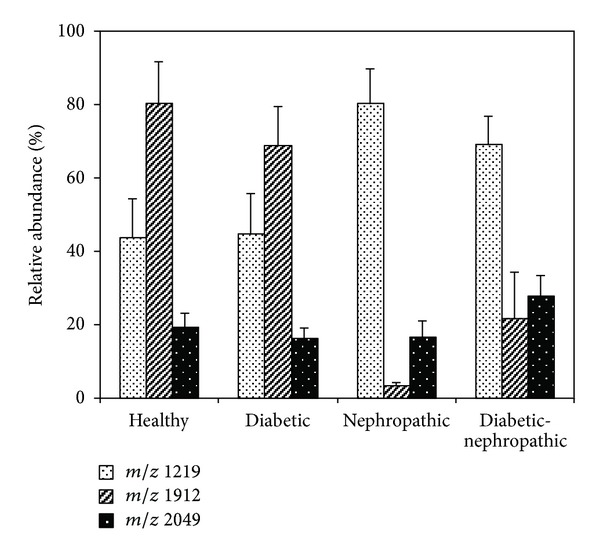
Histograms of the abundances of ions at *m*/*z* 1219, 1912, and 2049 as found in urine samples of subjects under study. Data are expressed as mean ± SEM.

**Figure 11:**
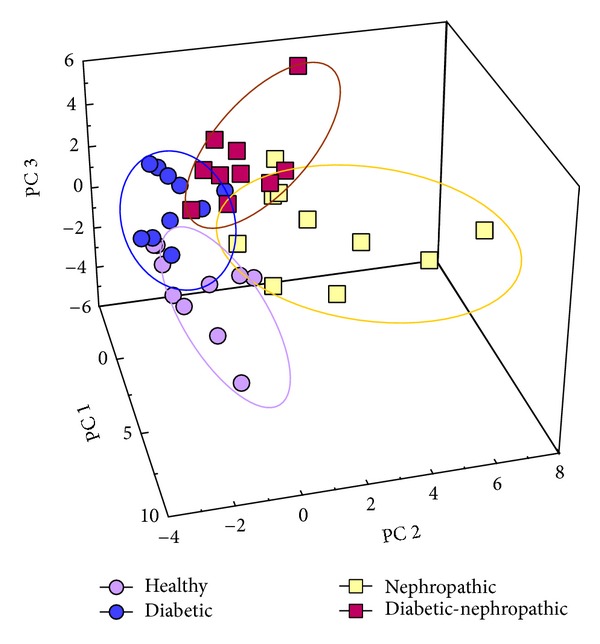
3D plot of the first three components resulting from the principal component analysis (PCA) of the MALDI spectra of patients and controls, using data that were statistically different at ANOVA (*P* < 0.05). The ovals indicate the regions of the principal component space containing data related to the different populations (healthy subjects as well as diabetic, nephropathic, and diabetic-nephropathic patients). From [42], by kind permission of Wiley and Sons through RightsLink, License no. 3064280001291.

A different approach was employed for the same aim, based on the identification of molecular species by their direct analysis, that is, without their tryptic digestion [42]. Urine samples from ten type 2 diabetic patients, ten patients affected by renal disease, ten diabetic patients affected by renal disease, and ten healthy controls were evaluated by a simple sample treatment and MALDI analysis of the low molecular weight peptides profile. Multivariate analysis suggested the possibility of a distinction among the considered groups of patients (Figure 10). Some differences have been found in particular in the relative abundances of three ions at *m*/*z* 1912, 1219, and 2049 (see Figure 11). For these reasons, a further investigation was carried out by MALDI/TOF/TOF to determine the sequence of these peptides and, consequently, to individuate their possible origin. By this approach, the peptide at *m*/*z* 1912 was found to originate from uromodulin, and its lower expression in the case of nephropathy can be well related to the pathological condition. Ions at *m*/*z* 2049 and 1219 originate from the collagen alfa-1 (I) chain precursor and from the collagen alpha-5 (IV) chain precursor, respectively, and, also, in this case, their different expressions can be related to the pathologies under investigation. Also, these data seem to indicate that urine is an interesting biological fluid to investigate on peptide profile and to obtain, consequently, information on dysmetabolism activated by specific pathologies.

Very recently, we compared the performance of CE-MS and MALDI-MS in detecting CKD [43], based on a cohort of 137 urine samples (62 cases and 75 controls). Data cross-talk between the two platforms was established for the comparison of detected biomarkers. The results demonstrate superior performance of the CE-MS approach in terms of peptide resolution and obtained disease prediction accuracy rates. However, the data also demonstrate the ability of the MALDI-MS approach to separate CKD patients from controls, at slightly reduced accuracy, but substantially reduced cost and time. As a consequence, a practical approach can be foreseen where MALDI-MS is employed as an inexpensive, fast, and robust screening tool to detect probable CKD. In a second step, high resolution CE-MS could be used in those patients only that scored positive for CKD in the MALDI-MS analysis, reducing cost and time of such a program.

## 9. Conclusions

The data reported in this paper show that MALDI-MS can be considered a valid analytical tool to study the glycation processes of proteins occurring *in vivo* conditions, which are relevant in presence of highly glucose concentration as in diabetes. Glycated proteins show necessarily a different functionality, and consequently their glycation level can give account for the long-term diabetic complications. When applied on biological fluid, the method allows to evaluate the presence of either glycation or oxidation stress and to determine biomarkers of specific diseases.

## Figures and Tables

**Table 1:** Metabolic data (fasting plasma glucose level, HbA1c %, furosine) relative to badly controlled diabetic patients, well-controlled diabetic patients, and healthy subjects.

Subjects	Fasting plasma glucose level (mmol/L)	HbA1c (%)	Furosine (*μ*g/mg protein)
Badly controlled diabetic patients	20.2 ± 4.3^a,b^	10.6 ± 1.9^a,b^	0.47 ± 0.08^a,b^
Well-controlled diabetic patients	7.96 ± 1.1^c^	7.25 ± 0.63^c^	0.33 ± 0.03^c^
Healthy subjects	5.46 ± 0.4	5.57 ± 0.43	0.23 ± 0.02

^a^ *P* < 0.001 compared to healthy subjects.

^b^ *P* < 0.001 compared to well-controlled diabetic subjects.

^c^ *P* < 0.001 compared to healthy subjects. Data are mean ± SD.
